# Why are individuals tracing travel trends? A case study of City Walk in Malaysia

**DOI:** 10.1371/journal.pone.0309493

**Published:** 2025-02-10

**Authors:** Zhenbin Wang, Hui Zhang, Sridar Ramachandran, Suiying Cheng

**Affiliations:** 1 School of Business, Anyang Institute of Technology, Anyang, Henan, China; 2 School of Accountancy, Anyang Institute of Technology, Anyang, Henan, China; 3 School of Business and Economics, Universiti Putra Malaysia, Selangor, Malaysia; 4 School of Culture and Tourism, Henan University, Kaifeng, Henan, China; Xiamen University Malaysia, MALAYSIA

## Abstract

Studying the emerging travel trends of City Walk is a beneficial activity for young groups. However, there is a lack of research and understanding regarding the motivation and mechanism behind these trends, both in theory and practice. The aim of this study was to investigate the motivation of persons who follow the travel trend of City Walk and evaluate how behavioral intentions are formed by exploring the link between motivation and behavioral intention using the self-determination theory, and social influence theory. Social influence, variety seeking, and self-identification were extrinsic and intrinsic motivations of behavioral intention. A quantitative purposive survey approach was employed, wherein 315 young individuals aged 18 to 40 were recruited to respond. The findings derived from the partial least squares structural equation modeling demonstrate that extrinsic incentives related to social influence, variety seeking, and health care have a considerable impact on behavioral intention, and to some extent influence self-identification. Self-identification has a mediating role in the relationship between health care and behavioral intention. By examining both theoretical and practical aspects, it seeks to provide useful theoretical insights and practical contributions to advance research and industry in the field of rural tourism.

## Introduction

Variety-seeking behavior is inherent to human nature [1] and has a substantial role in influencing tourists’ choices of travel locations, regardless of their level of satisfaction with previous experiences [2]. As tourism continues to grow and people increasingly prioritize high-quality experiences, there is a growing inclination to explore new types of tourism styles and seek a variety of experiences. This includes cultural tourism, heritage tourism, rural tourism, urban tourism, dark tourism, and more. Engaging in diverse experiences is beneficial as it helps reduce boredom and increase stimulation levels in our daily lives [3]. The Mckinsey poll of 2024 indicates that travel is a crucial goal for younger generations, who are eager to journey abroad and readily share experiences while drawing inspiration from social media. Consequently, forecasting and aligning with the emerging trends in tourist growth while offering appropriate goods for the younger generation seems to be the pivotal aspect of transformation in the future.

City Walk, as a popular travel trend, has drawn numerous people who seek to explore and experience urban settings. It has become a significant tourism destination for these travelers around the world. In a poll conducted in 2023, it was found that 82% of respondents expressed a desire to experience the city stroll, surpassing the popularity of all other travel modes in China (https://new.qq.com). The rapid increasing of city walk represents an opportunity for tour guides and travel service providers to offer a more tailored, professional service to meet ever-changing market demands (China Daily, 2023) which perfectly reflects the desire for variety seeking in tourism of people such as camping or glamping [4], heritage tourism or virtual reality experiences immersing [5,6]. City walks, highlights individualized experiences, integrate leisure and tourism functions, connecting various tourism and leisure activities such as shopping, eating, sightseeing, education, health, and variety-seeking.

A survey report on pre-travel outbound activities in Malaysia for 2024 indicates that individuals engaging in international holidays are influenced by factors such as budget constraints, social influence, word of mouth, activities, and experiences (https://data.tourism.gov.my). Consequently, apart from economic factors, social influences, activities, experiences related to health, and distinct current potentialities impact their future travel behavioral intentions. Previous noted that, visitors consistently generate and adhere to novel tourist fashions or trends in various settings, and the mass market is dividing into several specialized markets in response to the diversity of consumer preferences and demands [7]. The proliferation of social media, internet, and smartphones has facilitated the emergence of new consumer consumption habits [8]. These habits have a significant impact on others through bandwagon effects, which are closely related to people’s daily lives and their behavioral intentions in the tourism context.

As City walks is becoming a prominent travel trend and a favored option for tourists. research in this domain is paramount. The bulk of prior studies focused on the walkability of urban environments [9,10] or academic campuses [11], which often include designated sites such as shopping centers [12,13]. There is no exact description for this phenomenon in the realm of contemporary tourism as well. The fundamental reason for the heightened involvement of contemporary persons, especially the young demographic, with city walking as a fashion statement has been neglected. No study has been undertaken to investigate the fundamental causes that may elucidate why individuals are increasingly engaging in this conflicting circumstance. The motivation for monitoring emerging travel trends and the rationale behind consumers’ preference for adopting new fashion and enthusiastically investigating novel commercial avenues remain limited.

Previous studies considered that Self-Determination Theory (SDT) is essential to possess a thorough understanding of visitors’ motivation [14,15] which places significant emphasis on human motivation and personality, examining the extent to which individuals possess self-determination with empirical methods. However, the application of SDT in the tourist business, namely in city tourism sectors, has been largely overlooked, despite its potential in marketing and sales research. as well as Social Influence Theory (SIT), Which is a theoretical framework that analyzes how people’ thoughts, feelings, and behaviors are affected by the presence or actions of others. It posits that people, as constituents of a society, are inherently situated to either exercise social influence or be influenced by others [16]. It may be seen as a manifestation of how society seeks to mound an individual’s ideas, perceptions, attitudes, and behaviors via social interactions that encompass both positive and negative effects [16]. Nevertheless, there is a scarcity of research on the utilization of Social Influence Theory to investigate emerging tourist phenomena, particularly in relation to the recent trend of city walks.

As one of the most popular tourist destinations in Asia, Malaysia has rich cultural heritage, distinct local culture, and stunning natural monuments. The government created the National Tourism Policy 2020–2030 to sustain the country’s tourism sector and to position Malaysia among the top 10 worldwide tourist destinations. The Tourism Malaysia strategic plan (2022–2026) indicates that the Malaysian tourism industry is undergoing transformation, adopting new directions in both domestic and international tourism to enhance its appeal and competitiveness through diverse tourism types, incorporating elements of leisure, education, health, halal, and the concept of a second home. Meanwhile, the Visa exemptions in the APAC region for Chinese travelers would certainly enhance tourism in countries such as Thailand, Singapore, and Malaysia. For instance, outbound travel from mainland China, now at 80.3% of 2019 levels are the third biggest demographic of visitors in Malaysia, Singapore, and Indonesia, behind Malaysian tourists. While the younger generations exhibit a strong enthusiasm for foreign travel in the world, individuals aged 25 to 39 constitute the predominant proportion of overseas visitors in Malaysia. These travelers are part of the global workforce, possessing both the financial means and excellent health to undertake international journeys.

Therefore, this research aims to use social influence theory and self-determination theory to examine the dynamics of City Walk activities, the motives for individual engagement in city walks, and their relationship to behavioral intention. The objective is to formulate an academic definition of City Walk and establish a theoretical framework utilizing Self-Determination Theory (SDT) and Social Identity Theory (SIT) to analyze the interaction of these elements within the realm of city tourism. This will be approached from both theoretical and practical perspectives to achieve an in-depth comprehension of the motivations and mechanisms influencing individuals to adhere to travel trends and to devise strategies for future city tourism.

## Literature review and hypotheses development

### City work in the tourism context

Prior research has acknowledged that the concept of city walking originated in London, UK at the start of the 21st century. This was accompanied by the release of important policy documents in 2004, such as the UK walking and Cycling: an action plan, and the Capital’s walking plan for London. These plans were subsequently promoted by various interest groups through events, art, and other appealing activities, adding new significance to the concept [17]. City walk is a term used to describe certain physical establishments such as commercial streets, events, or resorts in several countries like the USA, UK, Dubai, Italy, and Indonesia [10,15]. Clearly, it is unable to elucidate the inherent nature of city walk within the framework of tourism. The focus on tangible elements, particularly in the age of widespread tourism, has restricted the perception of urban promenades. Walking is a primary means of transportation for many individuals and has been found to have a significant beneficial impact on mental and physical health. Additionally, it offers advantages for urban design and planning in creating walkable communities [18,19]. Thus, in this study, we have defined city walk as a recreational activity including strolling across metropolitan regions to acquire certain experiences while engaging in behaviors that seek diversity, all in a healthy manner. The sort of City Walk can encompass several goals and categories, including consumption, relaxation, diversity seeking, and historical exploration. City Walk offers a healthy and unique approach for both locals and tourists to explore, with places that are distinct from everyday situations. For instance, if you engage in a daily post-dinner walk within your residence, it cannot be considered a city walk. City Walk primarily caters to unconventional aspects of daily life, offering a range of activities that extend beyond typical tourist. It serves as a means of escape, enthusiasm, entertainment, education, and recreational purposes.

### Self-determination theory

Numerous travelers lack awareness of the exact definition of a new travel trend, and others may not even exhibit interest in its particular elements. Nonetheless, they see the new fashion as a substantial option, notwithstanding their uncertainty over trip plans due to the impact of prevailing travel patterns. Engagement in substantial physical activities profoundly influences personal identity and social connections [20]. The present study expands upon self-determination theory (SDT), initially proposed by American psychologists Edward L. Deci and Richard M. Ryan in the 1980s, by integrating it with social influence theory (SIT) to achieve a comprehensive understanding of visitors’ motivations and the factors influencing their behavioral intentions and the mechanisms involved [21]. The Self-Determination Theory encompasses elements like external regulation, intrinsic regulation, introjected regulation, and identifiable regulation [22]. The mechanism is a theoretical concept that posits the existence of intrinsic or self-determined incentives alongside extrinsic or externally regulated motives [23]. It suggests that people have an inherent tendency for self-integration, growth, and knowledge acquisition, which is fostered by the support received from their social and external contexts [24,25]. For example, Self-Determination Theory (SDT) has been used with anticipation theory and committee theory to examine internal banking users’ intentions to continue using online banking services [26].

Furthermore, SDT refers that Individuals are born with the tendency for psychological growth and integration. They can actively participate, actively absorb information and behavioral norms, integrate with social groups, and improve themselves. Individuals could engage actively, acquire knowledge and adopt behavioral standards, assimilate into social circles, and enhance their own abilities. According to research on tourism, different forms of motivation result in varied levels of tourist involvement and a range of results [19,27]. The SDT model has been used in tourism research, specifically to ascertain the motives for tourist participation among persons with disabilities [23]. Previous research corroborated the beneficial impact of attitude and perceived visitors’ desire to participate in tourism activities, influenced by people’ intrinsic cognitive beliefs [28]. This research examines how external environmental factors, including social influence, healthcare, and variety seeking, shape external regulation, which then impacts the development of intrinsic regulation of self-identification in tourism and affects behavioral intentions in city walking. The use of SDT in the City Walk research would provide a thorough comprehension of the motivations driving the developing travel trend of City Walk. The use of Self-Determination Theory in City Walk’s motivation research may facilitate a comprehensive understanding of the emerging travel trend.

### Social influence theory

Social Influence Theory (SIT) is a theoretical framework that examines how the ideas, emotions, and actions of individuals are influenced by the presence or behavior of others. SIT explores the influence of social variables on tourists’ decision-making processes and travel behaviors in the field of tourism. Travel trends may be seen as a form of fashion within the tourism industry. They represent a shift in visitor behavior and preferences, as well as changes in marketing strategies to attract tourists. Prospective groups often emulate or adopt fashion trends to satisfy their societal and personal aspirations Boto-García & Baños-Pino (2022), observed that individuals imitate the actions of others due to social conventions, ambitions, and the desire to conform to the people they want to be connected with [29]. Self-categorization theory suggests that an individual’s behavior is influenced by either societal or personal identification processes [30]. For visitors, following a travel trend may be a practical and effective approach when faced with uncertainty in making travel decisions. Individuals often engage in a process of observing the behaviors of others before making their own choices, depending on the outcomes they have witnessed [31]. Social influence is often categorized into normative social influence and informational social influence, both of which encourage people to comply. This research examines two scopes of social influence: the micro level, which pertains to the precise impact of social media, platforms, and real-world society. Previous research indicated that tourism habits and locations are increasingly shaped by the viewpoints of trusted peers via social media, where travel-related activities and information are disseminated [32]. Another aspect pertains to the macro scale, including societal expectations of people and their own aspirations. This research examines a broad perspective on social influence, characterized by extrinsic factors, using three indicators: social influence, variety seeking, and healthcare. However, previous research has mostly examined social impact via the lens of social media or standards, with few considering it as a broader perspective, particularly in the context of travel fashion monitoring, which remains limited.

### The hypotheses development

#### Social influence has effect on self-identification and behavioral intention.

The social influence is complex and multifaceted [16], which is challenging to explain and quantify. It undeniably exists in our everyday lives and has an impact on individuals both psychologically and physiologically. When used to track travel patterns in search of fashionable vacation destinations, it might indicate the bandwagon effect within the tourism industry. The “bandwagon” effect in economics refers to the phenomena where the demand for a product rises as a result of its popularity [8]. The “bandwagon” effect in marketing refers to the phenomenon where consumers adopt the same behavior and attitudes as a group or class they wish to associate with [8,33], and the field of social psychology, social behavior refers to the higher likelihood of an action occurring when individuals adopt a certain thought or behavior [8]. Conspicuous consumption occurs when consumers’ assessment of a product’s worth is influenced by its popularity [34]. The “bandwagon” effect in marketing refers to the phenomenon where consumers adopt the same behavior and attitudes as a group or class they wish to associate with [8,33].

Consumers exhibit bandwagon behavior to get social approval or project a fashionable image within their reference group [34]. Consumers may wish to communicate various aspects of their circumstances or character to others [35]. while enthusiasm to use social media as a main channel to share their travel experiences to enhance social visibility, which is a precondition for conspicuous consumption [36], that could lead to bandwagon effect in specific groups or social class. Because a signaling experience can be considered as variant of conspicuous consumption with high visibility of conspicuous behavior through rapid development of social media [36]. The proliferation of social media has expanded people’s opportunities to communicate and get information from many sources, therefore broadening their reference group beyond what was possible in the past [29].

The phrase “conspicuous consumption” emerged in the last century, transitioning from the economic realm to social contexts. It refers not only to the display of riches, but also to the manifestation of intangible symbolic elements [35]. The emergence of travel trends accompanied by new fashion may be seen as a form of intangible conspicuous spending that is influenced by the bandwagon effect in society. This phenomenon is closely associated with social status, money, and the need to demonstrate one’s identity. Studying travel patterns and trends may assist individuals in expressing their individuality and showcasing their achievements and social standing [29], as well as establishing a sense of self-identity that distinguishes them from others. Therefore, in this study, conspicuous consumption under the bandwagon effect is defined as the manifestation of social influence. Prior studies have noted the influence of conspicuous consumption, for instance, a conspicuous leisure study indicated that people not only visit a cultural event because of the quality of what is offered, but also to show something of their personality to relevant reference groups [36]. As cities have become the great centers of consumption, conspicuous consumption in urban context is more sensitive than inconspicuous consumption. However, limited studies have presented the research process on it in urban context, especially with new travel trends, such as City Walk. Thus, this study assumed that:

HI. Social influence has positive effect on individual’s self-identification.
H2. Social influence has positive effect on individual’s behavioral intention.

#### Variety seeking has effect on self-identification and behavioral intention.

Variety seeking behavior is part of human nature in any product of service consumption situation [1]. Previous research suggests that variety seeking behavior is driven by a desire to reduce boredom and increase stimulation, both intrinsically and extrinsically motivated. This behavior has been found to impact decision-making satisfaction [2] and is strongly associated with the intention to revisit and engage in word-of-mouth communication. The social learning theory posits that individuals’ behavior is influenced by the interplay between people and events, allowing for the acquisition of knowledge and skills through the observation of others within a social context [37], intrinsic and extrinsic motivations can both be influenced by social influence. Variety seeking behavior, on the other hand, can be seen as a manifestation of social learning behavior. It arises from the ongoing interaction between personal or cognitive factors (such as beliefs, attitudes, knowledge, skills, and self-efficacy), behavioral factors, and environmental factors (such as the learning environment, social norms, social motivation, incentives, and penalties) in reciprocal patterns [37]. Assessing travel trends and fashion may be seen as a manifestation of variety-seeking behavior, in contrast to conventional forms of travel such as rural tourism, historical tourism, and cultural tourism. Previous research indicated that variety-seeking behavior substantially affects tourists’ choices of destinations, especially historic sites [2]. The influence of intrinsic and extrinsic variety-seeking motives on pleasure with new restaurants subsequently revealed revisit intention, word of mouth, and connection to the establishment [1]. Despite the significance of variety seeking in relation to both extrinsic and intrinsic regulations and behavioral intentions, there remains a paucity of research on the phenomenon of variety seeking behavior within the realm of city tourism to explore the interaction between intrinsic and extrinsic regulations and behavioral intention. Consequently, the following assumptions were formulated:

H3. Variety seeking has positive effect on individual’s self-identification.
H4. Variety seeking has positive effect on individual’s behavioral intention.

#### Healthcare has effect on self-identification and behavioral intention.

Healthcare is a crucial element influencing an individual’s travel decision-making process. Typically, healthcare encompasses both mental and physical health, as per the World Health Organization. Mental health is a condition of psychological well-being that allows individuals to manage life’s stressors, recognize their capabilities, engage in effective learning and job, and contribute to their society. Physical health refers to the condition of the body and its functionality, namely the absence of sickness or illness. These two types of health are very increased. Subpar mental health may deplete your energy and hinder your physical health endeavors; nevertheless, enhancing your physical exercise can positively influence your mental well-being. Consequently, individuals are progressively predisposed to embrace health-oriented travel practices to improve their total well-being, including both mental and psychological dimensions. Zhang et al. (2021) demonstrate a significant link between location and health in health-oriented seasonal tourism, comprising both mental and psychological dimensions [38]. Recent studies increasingly recognize the essential relationship between location and well-being, since location is pivotal in addressing health-related issues [38].

Health is the result of an individual’s positive engagement with their perceptions of a certain region and its physical, social, and symbolic dimensions [34,35]. Tourism enhances mental well-being. Numerous mental health interventions need sustained behavioral modification [39]. Enhanced awareness of healthcare’s influence motivates customers to choose for better transportation methods or travel expressly for healthcare needs. Health tourism destinations are sites where customers engage with stakeholders to get beneficial health promotion materials [38]. Locations providing a pristine environment with clean air, wholesome food, uncontaminated water, and scenic vistas have gained popularity among health-conscious persons. These locations include national parks, countryside regions, and untamed landscapes [40–42]. Different ages significantly influence the physical and mental health advantages of visitors engaged in rural tourism activities [43].

Additionally, activities such as floating [44], skiing [45], hiking [45], camping [46] etc. put one’s eyes on health is also the trends in tourism niche marketing with rapid growth [47]. The emerging travel trends of slow tourism, city walk, and Special Forces-style tourism all promote a healthy approach to travel, offering a harmonious blend of experiential exploration and well-being. For instance, in urban settings, past research has suggested that walking is a primary means of transportation for a significant number of individuals This method of travel has been shown to positively influence both mental and physical health, while also fostering self-identification and shaping future behaviors. Individuals derive satisfaction from their investment of time and money in holiday travel, anticipating enjoyment that benefits their mental health, which subsequently impacts their physical well-being [39]. Consequently, leisure tourists, who invest in their mental health, ultimately create an independent economic contribution through alterations in social behavior [39]. Notwithstanding the environmental and health significance of pedestrian tourism, dedicated research on walking within this setting is scarce, with leisure and occupational walking often serving as a proxy [48]. Therefore, the correlation between “healthcare and individuals’ self-identification and behavioral intent.

H5. Health care has positive effect on individual’s self-identification.
H6. Health care has positive effect on individual’s behavioral intention.

#### Self-identification and behavioral intention.

The concept of self-identification has received much attention in the field of marketing [49], It refers to the process by which customers fulfil their psychological requirements by associating themselves with a particular location [50]. Self-identification, as described by previous research, refers to the degree to which a visitor believes that a tourism location aligns with their own personality [49,51]. Kahraman & Cifci (2023) revealed that self-identification positively affects overall satisfaction, memorable tourism experiences and destination loyalty which would lead to potential behavioral intention in tourism context [50], as well as Alrawadieh et al. (2019) investigated that self-identification has positive effects on overall satisfaction with a heritage tourism site [49]. Tracing travel trends or fashion, such as city walk, can be an activity that allows visitors to connect and interact with society from new perspectives. This process can contribute to positive self-identification and ultimately lead to overall satisfaction and behavioral intention in a holistic manner. Therefore, the study proposed that:

H7. Individual’s self-identification has positive effect on individual’s behavioral intention.

#### The mediation role of self-identification.

Self-identification is seen as a significant intrinsic regulation that reflects extrinsic regulation. Prior research has identified belongings across many categories, including ecotourists, groups with distinct features, and Generation Z. For example, the expanding impact of ecotourism results in a greater number of conventional tourists adopting the identity of ecotourists, demonstrating heightened environmental awareness and responsibility [52]. Additionally, research on dietary behavior reveals that individuals who actively participate in vegan activism tend to possess stronger moral convictions, heightened collective anger, and a stronger sense of identification [53]. This suggests that moral convictions, as a form of social influence, can affect self-identification and subsequently lead to intentions for environmentally conscious behavior. The increase in research concerning environmental concerns within the travel context has shown that those who identify as environmentally conscious have inclinations to embrace electric aircraft [54]. The same significance is also evident in other domains, such as healthcare. Previous research shown that women exposed to a memorable message reported a markedly higher frequency of days per week dedicated to good food and exercise activities, particularly influencing health outcomes among Black women [55]. Self-identification serves as a mediator connecting health-seeking and health-maintaining behaviors. Moreover, increased engagement in diverse tourist activities fosters a perception of distinct travel groups characterized by distinctive norms or regulations, which are also influenced by the self-identification between novelty-seeking and behavioral intention. For example, backpacking, as a distinctive form of tourism that captivates a growing number of persons, is associated with participants who self-identify as backpackers being less inclined to participate in unsustainable practices [56]. Moreover, self-identification has been seen as an effective approach to address tourism development challenges related to attracting visitor participation. In Laksana Village, homestay tourist managers identify difficulties to provide the foundation for the CS Team to delineate the nine components of the canvas business model [57]. Prior research has demonstrated that self-identification serves as a crucial intrinsic factor in examining behaviors and behavioral intentions across various domains, including tourism. However, its application in the context of city tourism studies, particularly in the investigation of travel fashion tracing, is insufficiently addressed. Consequently, this study posits the mediating effects of self-identification as follows:

H8. Individual’s self-identification mediates the relationship between social influence and behavioral intention.
H9. Individual’s self-identification mediates the relationship between variety seeking and behavioral intention.
H10. Individual’s self-identification mediates the relationship between health care and behavioral intention.

## Methods

### Measurement instrument

**Fig 1:**
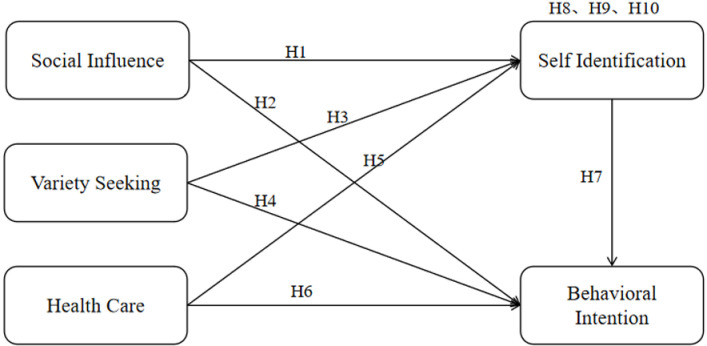
The concept of proposed model.

The study has been approved by Anyang University ethics committee, and respondents had been informed written before the survey. The positivist research paradigm in line with deductive reasoning is employed to test both the theories and hypotheses. A proposed model (Fig 1) was evaluated using a qualitative survey methodology. Questionnaire for the study was created consisting of two separate sections: one for collecting demographic information and the other for measuring constructs using multiple-item scales that were modified from earlier research. The primary components of social influence encompassed the two modalities of normative social influence and informational social influence. These dimensions are enabled by prior study supported researcher to deep understand the social influence. Jang et al. (2024) presents an integrative framework, emphasizing the pivotal role of the learning process, particularly when combining two distinct social influences of normative social influence and informational social influence for senior technology acceptance [58]. Oliveira et al. (2020) investigated the reason why people share their travel experiences on social media under social influence theory and mentioned that social media is becoming increasingly important for the tourism industry and there are facilitators and inhibitors regarding the practice of sharing travel experience on social media [32]. Therefore, this study drawing from prior studies in two dimensions of normative social influence and informational social influence with 8 items.

The Human beings naturally exhibit variety-seeking behavior while consuming any goods or service [1]. Previous research indicates that variety-seeking behavior is driven by a desire to reduce boredom and increase stimulation. Both Lee et al. (2020) and Van Trijp et al. (1996) have mentioned that intrinsic and extrinsic motivations are the influencing factors of individuals’ variety -seeking behaviors [1,59]. Lupu et al. (2021) investigated that information search as well as variety seeking behavior significantly influence the visitors’ selection for any tourist destination [2]. Taylor et al. (2018) investigated the moderating role of variety seeking behavior in pop-up dining experiences with 8 items (translated from Dutch) which based on Van Trijp & Steenkamp (1992) consumers’ variety seeking tendency with respect to foods [60,61]. Thus, in this study, 8 items were employed. Health care mainly contains psychological health and mental health. psychological health. Prior studies used different measurement to measure the health of body and mind. Mental health items often derive from well-being and happiness measures used in tourism research, focusing on feelings of relaxation, contentment, and reduced stress. Physical health items draw on measures of physical activity, pain reduction, and overall physical fitness improvements noted in tourism and health studies [38,62]. Therefore, in this study, health care includes two dimensions physical health and mental health, each dimension has 7 items.

Measurement for self-identification. Alrawadieh et al. (2019) accessed a conceptual model that postulated relationships between self-identification with a heritage site, engagement at the site, overall satisfaction and destination loyalty with 8 items [49]. Kahraman & Cifci (2023) investigated the influencing factor of self-identification in empirical study with seven items [50]. Considering some of the questions may be similar to each other, In this study, 6 items were applied to make more efficient understanding of the role of self-identification. Behavioral intention in this study had three dimensions: intent to return, word of mouth, and recommendation [63], assessed using three questions. The demographic profile section was intended to gather information on the sample’s attributes, including age, gender, educational attainment, and income level.

Four experts from the tourist, leisure, and hospitality sectors, together with 35 participants, were asked to engage in the pre-testing and pilot testing to ascertain the reliability and validity of the questionnaire. Ultimately, Social Influence including 8 questions, Variety Seeking consisting of 8 items, Health Care having 7 things, Self-Identification featuring 6 items, and Behavioral Intention encompassing 8 items were used to construct Section 1 of the questionnaire (Table 1).

**Table 1:** Measures and scales.

Construct	Source(s)	Items
Social influence	Jang et al. (2024); Oliveira et al. (2020) Taylor et al. (2018); Lupu et al. (2021)	8
Variety seeking	So et al. (2014); Rasoolimanesh et al. (2021)	8
Health care	Chen & Petrick (2013); de Bloom et al. (2012) Nawijn & Mitas (2012); Qin & Zhang (2018)	7
Self-identification	Kahraman & Cifci (2023); Alrawadieh et al. (2019)	6
Behavioral intention	Hollebeek (2021)	3

### Study setting and sample

Malaysia is one of the famous tourism destinations. It renowned for its abundant tourism resources. Malaysia boasts a diverse array of attractions, ranging from picturesque beaches and lush forests to excellent hospitality services and captivating city tours, catering to a wide spectrum of travelers. This diversity is attributed to Malaysia’s advantageous geographical location and the richness of its tourism services. According to the Malaysia Tourism Key Performance Indicators for 2022, 16.4% of visitors came to Malaysia for the purpose of holiday, leisure, relaxation, or simply getting away, while 29.9% visited for shopping. Also, based on statistics from Tourism Malaysia, the country received 20.14 million international arrivals and about 208.4 million domestic visitors in 2023. The target population of this research consisted of domestic and international visitor data from Malaysian government reports. The minimal sample size was established using two tools: G*Power software and the Raosoft Sample Size Calculator. These instruments were used to do mathematical calculations and ensure accuracy in the determination of the sample size. The Krejcie and Morgan table was used for verification purposes.

The United Nations defines youth as those aged 15 to 25 years. Numerous nations, particularly in the developing world, extend the age threshold for youth beyond the internationally acknowledged maximum of 25 years. In Malaysia, the National Youth Policy defines youth as those aged 15 to 40 years. In China, an individual is regarded as a youngster until the age of 45 years [64]. Thus, the young group was defined age between 18 to 40 years old.

### Data collection and analysis

The designed questionnaire survey was conducted to eligible respondents selected via purposive sampling based on the following criteria: 1) Visitors have engaged in City Walk for recreational purposes in Malaysia during the last six months; 2) Aged between 18 and 40; 3) Capable of articulating their experiences effectively. Data collection occurred by online and in-person surveys from May 10 to June 30, 2024. The respondents include local and international students aged 18 to 40 enrolled in Malaysian universities, including UKM, USM, UM, UITM, UPM, Taylor’s University, and Sunway University. The questionnaire was composed in both Chinese and English.

The Windows software SPSS 26.0, along with Smart-PLS 4.1, was utilized for data cleaning and analysis. SPSS offers a user-friendly platform for statistical analysis in social science and behavioral research [65]. PLS-SEM conducts analyses on latent constructs (unobserved) as well as manifest variables (observed) within the framework of structural equation modeling [66–68]. This approach is particularly valuable when dealing with relatively small sample sizes, non-normal data distributions, or measurement instruments characterized by low reliability. Consequently, 60 replies were discarded due to uniformity across all questions, despite the absence of missing data. A total of 315 people from various nations, including Malaysia, Singapore, China, Algeria, Bangladesh, India, and Indonesia, were compiled for additional analysis in the final database. The research used a two-stage approach, using the repeated indicator method, to analyze the measurement model including both first order and second-order components. PLSpredict was considered the most direct method for evaluating the predictive capabilities of the PLS path model.

## Results

### Sample characteristics

Out of 375 respondents, 315 participants were selected for the study, representing 84% of the total sample. The majority of respondents were female, accounting for 75.9%. A substantial proportion of participants (81.6%) were aged between 18 and 32 years, with the largest subgroup being those aged 18 to 22 years, comprising 35.9% of the sample. In terms of educational attainment, the majority had completed at least a bachelor’s degree or higher, with bachelor’s degree holders representing the largest group at 46.7%. Regarding the frequency of participation in City Walk activities, 34.3% reported engaging nonscheduled, followed by occasional participation (20.6%) and weekly participation (18.4%). In terms of duration, 52.1% of respondents typically engaged in City Walks for 1 to 3 hours, while 28.9% participated for 4 to 6 hours. Additionally, 70.2% of respondents participated in City Walks with friends, followed by family members (18.1%) and solo participation (9.8%).

### The measurement model

Reliability and validity were tested before establishing the measurement model, KMO value was 0.96, Harman’s single factor technique was employed to evaluate the data with the principal component factor analysis which focuses on the first -factor explanation percent to the variance of 16.53%, while compared with the standard of below 50 percent.

In this study, indicators of social influence, healthcare first order in reflective and second order in formative, the repeated indicator technique approach was employed to assess the measurement model. Validation of the measurement model involved examining the validity and reliability of all latent variables (Table 2). The first-order latent variables were established by checking the indicator loadings and cross-loadings. Items VS5 (0.685) and VS7 (0.653) were removed due to a low loading value below 0.7. All remaining indicator loadings exceeded the recommended threshold. The Variance Inflation Factor (VIF) values for indicators in the first-order constructs were below 5.0.

**Table 2:** Assessment of measurement model.

Laten variable	Item	Outer loading (Before)	Outer loading (After)	Cronbach’s Alpha	CR	AVE
Behavioral intention	BI1	0.812	0.811	0.873	0.874	0.725
	BI2	0.872	0.872			
	BI3	0.883	0.883			
	BI4	0.838	0.838			
Mental health	HC1	0.797	0.802	0.883	0.884	0.682
	HC2	0.836	0.833			
	HC3	0.878	0.877			
	HC4	0.350	Delete			
	HC5	0.780	0.787			
	HC6	0.821	0.828			
Physical health	HC7	0.770	0.770	0.912	0.913	0.655
	HC8	0.790	0.790			
	HC9	0.794	0.794			
	HC10	0.875	0.875			
	HC11	0.804	0.804			
	HC12	0.790	0.790			
	HC13	0.836	0.836			
Self-identification	SEI1	0.838	0.838	0.897	0.900	0.662
	SEI2	0.793	0.793			
	SEI3	0.886	0.886			
	SEI4	0.824	0.825			
	SEI5	0.813	0.813			
	SEI6	0.717	0.716			
Normative social influence	SOI1	0.808	0.808	0.814	0.815	0.643
	SOI2	0.781	0.781			
	SOI3	0.849	0.849			
	SOI4	0.766	0.766			
Informational social influence	SOI5	0.832	0.832	0.844	0.845	0.682
	SOI6	0.808	0.808			
	SOI7	0.810	0.810			
	SOI8	0.852	0.852			
variety seeking	VS1	0.674	Delete	0.864	0.864	0.647
	VS2	0.759	0.781			
	VS3	0.808	0.823			
	VS4	0.766	0.793			
	VS5	0.612	Delete			
	VS6	0.792	0.811			
	VS7	0.591	Delete			
	VS8	0.801	0.814			

Cronbach’s alpha values ranged from 0.864 to 0.912, exceeding the recommended minimum level of 0.70, indicating satisfactory internal consistency reliability. Composite reliability values (CR) were also above the recommended limit of 0.7, ranging from 0.864 to 0.913, confirming the reliability of internal consistency. The Average Variance Extracted (AVE) values were greater than the threshold of 0.5, ranging from 0.643 to 0.725, verifying convergent validity. Discriminant validity of the first-order reflective constructs was assessed using both the Heterotrait-Monotrait (HTMT) ratio and Fornell-Larcker’s criterion. The majority of HTMT values were below 0.85 and 0.90. The discriminant validity has been established (Table 3).

**Table 3:** Discriminant validity- HTMT Matrix (first order).

	BI	NSI	SEI	VS	ISI	MH	PH
BI							
NSI	0.697						
SEI	0.819	0.692					
VS	0.755	0.628	0.727				
ISI	0.719	0.838	0.665	0.602			
MH	0.855	0.714	0.855	0.813	0.698		
PH	0.779	0.709	0.876	0.702	0.689	0.896	

Note: BI (behavioral intention), NSI (Normative social influence), ISI (Informational social influence), SEI (Self Identification), VS (Variety seeking behavior), MH (Mental health), PH (Physical Health).

Validation of the second-order constructs was based on the data derived from the first-order latent scores. Cronbach’s alpha values ranged from 0.822 to 0.897, while CR values ranged from 0.823 to 0.893, all above 0.7, both exceeding the recommended limits. AVE values ranged from 0.647 to 0.903, all above the 0.5 threshold. Thus, reliability and convergent validity were achieved. Discriminant validity has been effectively demonstrated, as indicated by all HTMT values being below 0.85 and 0.90, except for 0.914 which was slightly above 0.90 (Table 4). In addition, the empirical 95% confidence interval created through bootstrapping in Smart PLS does not contain the value of 1 (Table 5) and the majority of VIF values are below 3.1. This indicates that there is sufficiently discriminant validity.

**Table 4:** Discriminant validity- HTMT Matrix (second order).

	BI	HC	SEI	SOI	VS
BI					
HC	0.862				
SEI	0.819	0.914			
SOI	0.773	0.809	0.739		
VS	0.755	0.799	0.727	0.671	

Note: BI (behavioral intention), HC (Health Care), SEI (Self Identification), SOI (Social influence), VS (Variety seeking behavior).

**Table 5:** Confidence intervals (second order).

	Original sample (O)	Sample mean (M)	2.5%	97.5%
HC < - > BI	0.862	0.863	0.798	0.921
SEI < - > BI	0.819	0.819	0.745	0.883
SEI < - > HC	0.914	0.914	0.859	0.962
SEI < - > BI	0.773	0.774	0.686	0.852
SOI < - > HC	0.809	0.810	0.725	0.887
SOI < - > SEI	0.739	0.739	0.640	0.826
VS < - > BI	0.755	0.755	0.663	0.835
VS < - > HC	0.799	0.800	0.706	0.883
VS < - > SEI	0.727	0.727	0.630	0.815
VS < - > SOI	0.671	0.672	0.557	0.782

Note: BI (behavioral intention), HC (Health Care), SEI (Self Identification), SOI (Social influence), VS (Variety seeking behavior).

### The structural model

The results indicate that social influence has significant effects on self-identification and behavioral intention, H1(*β* = 0.112, *p* = 0.039), H2(*β* = 0.189, *p* = 0.001) were supported (Table 6). The construct of variety seeking has significant effect on behavioral intention, but there was no significant effect on self-identification. That means H3(*β* = 0.112, *p* = 0.053) was not supported while H4(*β* = 0.183, *p* = 0.003) was supported by the study. The results shows that health care have positive effects both on self-identification and behavioral intention, that means H5 (*β* = 0.663, *p* = 0.000), H6(*β* = 0.314, *p* = 0.000) were supported, as well as H7(*β* = 0.229, *p* = 0.000), indicates that self-identification has significant effect on behavioral intention. The results show that the mediation role of self-identification between health care and behavioral intention was significant, H10(*β* = 0.152, *p* = 0.001) was supported. However, the mediation role of self-identification between social influence, variety seeking, and behavioral intention were not significant, H8(*β* = 0.026, *p* = 0.089), H9(*β* = 0.026, *p* = 0.094) were not supported.

**Table 6:** Results of structural model.

Path	Original sample (O)	Sample mean (M)	T statistics	P values
H1.SOI - > SEI	0.112	0.111	2.066	0.039 *
H2.SOI - > BI	0.189	0.189	3.361	0.001**
H3.VS - > SEI	0.112	0.116	1.935	0.053
H4.VS - > BI	0.183	0.185	3.010	0.003**
H5.HC - > SEI	0.663	0.660	10.430	0.000***
H6.HC - > BI	0.314	0.311	3.988	0.000***
H7.SEI - > BI	0.229	0.232	3.534	0.000***
H8.SOI - > SEI- > BI	0.026	0.026	1.700	0.089
H9.VS - > SEI - > BI	0.026	0.027	1.673	0.094
H10.HC - > SEI - > BI	0.152	0.153	3.273	0.001**

Note: BI (behavioral intention), HC (Health Care), SEI (Self Identification), SOI (Social influence), VS (Variety seeking behavior).

*Means sig < 0.05,

**means sig < 0.01,

***means sig < 0.001.

**Fig 2:**
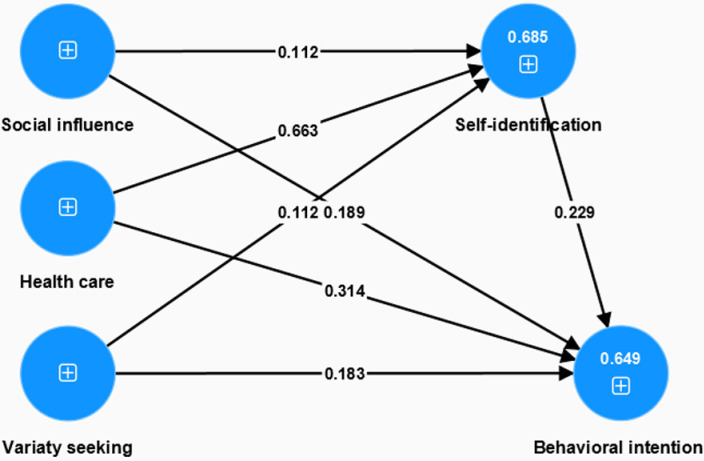
Results of structural equation modeling (PLS-SEM).

The R^2^ values indicated the strong explanatory power of the structural model (Fig 2), whereby self-identification accounted for 68.5% of the variance in behavioral intention and the motivation factors accounted for 64.9% of the variance in self-identification. Further, the large effect size, f^2^, of 0.528 shows that health care exhibited the most substantial influence on self-identification, followed by variety seeking and social influence.

PLSpredict, based on the concepts of separate training and holdout samples for estimating model parameters and evaluating a model’s predictive power [69], was employed in the second order. The results indicate that Q^2^_predict_ was greater than zero, ranging from 0.391 to 0.555, and PLS-SEM < LM for all indicators (Table 7); therefore, the model had high predictive power. Notably, the investigation of the mediating effect in this study revealed that self-identification plays a significant partial mediating role between the health care and behavioral intention, with H10 were accepted.

**Table 7:** PLSpredict assessment of manifest variables.

	Q² predict	PLS-SEM_RMSE	PLS-SEM_MAE	LM_RMSE	LM_MAE
BI1	0.432	0.592	0.442	0.599	0.435
BI2	0.487	0.524	0.393	0.535	0.398
BI3	0.467	0.562	0.416	0.575	0.425
BI4	0.412	0.557	0.437	0.565	0.440
SEI1	0.445	0.702	0.523	0.706	0.522
SEI2	0.391	0.681	0.484	0.687	0.484
SEI3	0.555	0.577	0.402	0.591	0.405
SEI4	0.482	0.622	0.462	0.630	0.462
SEI5	0.436	0.656	0.501	0.666	0.511
SEI6	0.365	0.881	0.691	0.878	0.662

Note: BI (behavioral intention), SEI (Self Identification).

## Discussion and conclusion

The present research analyzed the relationships between social influence, variety seeking, health care, self-identification, and behavioral intention within the framework of City Walk to explore the factors driving visitor tracking of travel patterns in the contemporary tourist business. A quantitative methodology was employed to ensure accuracy and comprehensiveness. The study has produced extensive findings with strong empirical evidence by including relevant factors into the research framework, gathering survey data from a representative sample, and used PLS-SEM to examine the structural connections among the variables. These findings give unique insights into the theoretical and practical knowledge in the area, providing a comprehensive grasp of how behavioral intention is impacted and created in City Walk activity.

### Discussion

The findings reveal that the independent constructs of social influence, variety seeking, and health care significantly affect behavioral intention. The findings align with prior research. Previous research demonstrates that social influence impacts individual choices about holiday trips and location selection [29], and it might enhance the acceptability of female users, particularly with the fast advancement of consumer self-service kiosks in hotels [70]. Consistent with previous research indicating that variety-seeking behavior is inherent to human nature in any consumption context [1], this behavior also serves as a means of establishing uniqueness. By pursuing diverse activities and experiences, individuals can distinguish themselves and categorize into different groups, thereby gaining experiential advantages over others. A research on the food variety-seeking behavior of Chinese tourists indicated that prevention-focused customers exhibit a decreased propensity to seek food diversity compared to promotion-focused consumers [71]. In addition to marketing, consumers’ propensity for variety seeking positively influences an individual’s desire to transfer services [72]. Previous shows that tourist destination in the context of healthcare and medical tourism can managed together with the study of the visitor consumer behavior [47]. Leisure tourists, who invest in their mental well-being, ultimately create an independent economic contribution via alterations in social behavior [39]. Consequently, within the expansive framework of social influence, the indicators of narrative social influence, variety seeking, and health care are substantial determinants of behavioral intention in the contemporary setting of tourism, such as city walk.

Secondly, only social influence and health care present significant effects on self-identification, there is no direct effect of variety seeking on self-identification, which was partially aligned with prior studies. previous studies has indicated that, in conspicuous leisure people not only visit a cultural event because of the quality of what is offered, but also to show something of their personality to relevant reference groups [36], Both mental health and physical health have effects on self-identification while recent studies are increasingly recognizing the vital links between place and health while place plays an essential role in addressing the issue of health [38]. People are rising to enjoyment they paid, when they spend money and time on holiday travel, they expect to enjoy themselves which is benefit to their mental health [39], and health tourism which highly relevant to medicinal treatment were popular in elder adults, while health care already be considered as a health way of leisure and suitable for both healthy individuals and the disables as a way of participating social activities. Contrary to prior studies suggesting that variety-seeking behavior is inherent to human nature in all consumption contexts [1], this research examines the influence of extrinsic and intrinsic variety-seeking motivations on satisfaction with new restaurants, which subsequently elucidates revisit intention, word-of-mouth, and attachment [1]. The findings of this research may be attributed to the accessibility of City Walk, which a majority of people can afford and engage in for many objectives, in contrast to particular leisure activities such as golf, personal service packages, and space tourism. The convenient accessibility of City Walk diminishes obstacles for visitors.

Thirdly. the results indicate that self-identification plays a mediating role in the link between health care and behavioral intention. there is no significant mediation impact between social influence and behavioral intention. The substantial mediating influence of self-identification may arise from individuals’ concern for both their mental and physical well-being. People who prioritize their health are more likely to adopt effective strategies to maintain their well-being and performance, which is strongly associated with self-identification. Nevertheless, social influence can manifest in various forms and situations. Not all social influence contributes to the awareness of self-identification; other forms of social influence are just suited for fulfilling momentary consumer needs. For instance, if someone is searching for a great restaurant to indulge in their favorite food, and another person happens to post their personal experience online, this type of social impact has minimal connection to self-identification. If an individual desires to choose a suitable location to showcase their social standing or accomplishments, they may align their self-identification with the prevailing trends influenced by society. The result may also be attributed to the kind of leisure and tourist activities in which visitors engage. When an activity or program has more precise needs that are highly pertinent to the social class category and presents distinctive distinctions in comparison to others, extrinsic regulation will transition to intrinsic regulation, resulting in an outcome. The research suggests that City Walk may function as a mass leisure initiative with less restrictions and constraints, resulting in diminished impacts on self-identification.

### Conclusion

The research seeks to examine the reasons people follow travel trends by integrating Self-Determination Theory (SDT) and Social Identity Theory (SIT) via an empirical study of City Walk in Malaysia. The research further enhances the comprehension of social influence via a micro lens focused on social media and platforms, as well as a macro perspective including narrative social influence, variety seeking, and healthcare, which function as forms of extrinsic control. Self-identification is seen as an internal regulation resulting from extrinsic regulation, which subsequently influences behavioral intention. The findings demonstrate that markers of social influence, variety seeking, and health care significantly affect behavioral intention, whereas self-identification serves as a mediator factor between health care and behavioral intention. The foundational assistance elucidates why people are inclined to analyze travel patterns from both practical and academic viewpoints inside City Walk, which would contribute to the future growth of urban tourism, particularly in Malaysia.

Firstly, Social impact in the macroscope must be meticulously evaluated while assessing and exploring emerging travel phenomena and trends in tourism and hospitality, given its substantial direct significance to behavioral intention. Tourists today more easily to be influenced by social influences through social media, trusted friends than ever before [32]. Therefore, constructing attractiveness social topic or travel waves with broad range of visitors through social medias, platforms such as face book, X, TikTok aims to differentiate categories of respondents is becoming an efficient way to promote tourism industry development and innovation promotion. City walk tend to be a strong travel trends in the world absolutely benefit from the development of social influence effects.

Furthermore, variety seeking constitutes the fundamental nourishment for humans, whether via novelty seeking, genuine seeking, or engagement seeking in diverse tourist or leisure activities, the primary aim of such experiences serves as the basis. The continued evolution of economic experiences, characterized by novelty, hedonism, enthusiasm, engagement, knowledge, and local culture, may serve as the primary allure for younger generations. This appeal is not solely for the acquisition of unique experiences but also for the opportunity to share them with others. In contrast to conventional urban tourist programs and events, City Walk integrates several elements of tourism, ranging from ancient architecture to contemporary design, and from traditional historical streets to current retail experiences. It offers visitors the chance to engage with ordinary life in cities, so extending the scope of tourism beyond designated sites to include the whole urban environment. Consequently, the amalgamation of leisure, tourist, and quotidian life that offers a more profound experience and comprehension of the city will be a focal point for future urban tourism growth.

Moreover, healthcare, which has already been recognized as an important influencing factors of tourism development. Health is experienced when a person’s perceptions associated with a particular place interact positively with the physical, social, and symbolic factors of a place [38]. Health tourism destinations are typical places of consumption in which consumers engage with actors to obtain beneficial health promotion resources [38]. Contemporary visitors are more inclined towards leisure or health-conscious travel, rather than incurring expenses on medical care. Nevertheless, prior research often associates health care tourism with medical treatment and experiences; few studies have examined the impact of healthcare on self-identification and behavioral intentions across other domains with a range of activities and programs. It restricted both recognition and implementation of health care in tourism. City walking is an emerging travel trend that promotes travelers engaging in leisure activities in a health-conscious manner, integrating leisure, tourism, and healthcare, while also enhancing the creativity of urban tourism.

Additionally, self-identification as intrinsic regulation was partially worked, the mediating effect of self-identification between social influence and behavioral intention was not significant, and there is no mediating effect between variety seeking and behavioral intention. The result reflects the awareness of health care of individuals both in mental and physical health, and the developing influence of social influence on people through technique ways with social media, online community, and platform. However, the supply of variety in City tourism is limited, the boundary of core tourism site is obviously berried the foot point of tourist to explore and experience an expending city area. City walk an integration of leisure and tourism allow tourists extending their food points to details of City, and deep involve in the daily life with a healthy path. Thus, future city tourism activities and programs should consider the importance relevance between City tourism sites and tourists’ self-identification, strengthen the linkage between extrinsic regulation and intrinsic regulation of tourist.

Ultimately, visitors want to immerse themselves in and participate in the local culture. Local experiences, such as savoring regional food and commemorating local festivals and holidays, are emerging as the foremost trends in tourism, with the amalgamation of leisure and tourism prompting more participation in everyday life. Diverse categories of leisure and tourist marketing drive innovation in city tourism, enhancing both pleasure and output. Consequently, developing an appealing brand for urban tourism via many channels, offering rich and distinctive experiences for tourists, and integrating healthcare with city tourism would constitute an effective strategy for the advancement of city tourism. Simultaneously, enhancing the connection between tourist destinations and tourists’ self-identification will facilitate the development of specific commercial opportunities. Previous research indicates that self-identification has been extensively examined in marketing (50), it develops when customers fulfill their psychological demands via affiliation with a place [50].

### Theoretical contributions

This research highlights the need of using a comprehensive approach based on the integration of SDT and SIT with influencing constructs of social influence, variety seeking, health care, and self-identification on behavioral intention when studying the motivation of persons who follow the travel trend of City Walk. This research is a groundbreaking attempt to empirically assess the links between the factors being studied, specifically within the context of City Walk. As a result, it contributes to the progress of scholarly discussion in this subject. Firstly, this study provides a theoretical framework by integrating SDT and SIT, for comprehending the development of visitors’ behavioral intention in City Walk and enhancing the research perspectives in this field. While prior studies have not extensively examined the emerging travel trend of City Walk, this study aims to provide a comprehensive explanation and exploration of the underlying processes of City Walk.

Moreover, this study enhancing and extending SDT and SIT by developing and analyzing a thorough theoretical framework that incorporates factors associated with social influence, variety seeking, healthcare, and self-identification. This study presents a validated framework that sheds light on the motivation in City Walk in the context of City tourism. It enhances our understanding of the complex relationships between social influence, variety seeking, health care, self-identification, and behavioral intentions. This study enhances the theoretical significance of the literature by emphasizing the role of self-identification in the context of city tourism. It deepens our understanding of how social influence and healthcare impact self-identification and, as a result, behavioral intentions.

Additionally, this research enhances the comprehension of variety seeking and healthcare in city tourism, particularly in relation to the emerging trend of City Walk. Previous research on variety seeking has primarily concentrated on novelty and authenticity in restaurants, leaving a dearth of implications for leisure and tourism, particularly regarding travel trends. This study broadens the implications of variety seeking to encompass travel trends within the leisure sector. Regarding the healthcare indicator. Prior research mostly focused on medical tourism and natural tourism within the healthcare framework; however, a limited number of studies acknowledged that both mental and physical healthcare are integral to leisure and tourist contexts, with little association to medical and natural environments. This research offers a novel viewpoint in the leisure and tourism sector, recognizing it as a significant element affecting persons’ travel habits.

### Practical implications

This study provides valuable insights and practical recommendations for managers, practitioners, and policymakers in city tourist destinations. By understanding visitors’ desire to follow travel trends and the elements that influence their behavioral intentions, these stakeholders may enhance the growth of high-quality tourism.

The research indicates that social influence, variety seeking, and health care are three crucial elements that drive the incentive to track the trip trend of City Walk among managers and practitioners. It highlights that these three aspects have a beneficial impact on behavioral intention and suggest that city tourism managers and practitioners should carefully examine social influence, continuously innovate, and offer a range of contexts to attract tourists. Examples include distinctive street architectures, themed streets offering local cuisine, cold-weather attire, souvenirs, and user-friendly directions that are easily accessible. In addition, it is important for them to not only adapt to the evolving interests of visitors, but also actively influence and shape visitors’ preferences using a combination of conventional and contemporary methods by utilizing social media platforms, professional websites, and other types of content such as short videos, storytelling, and live events. Moreover, it is crucial to acknowledge the strong correlation between travel programs and healthcare when individuals are seeking the most beneficial ways to spend their time in city tourist marketing. Greater emphasis needs to be placed on the integration of healthcare with leisure and tourist activities and programs, addressing both mental and physical health. Including healthy activities, immersive participation programs, tranquil experiences accompanied by gentle music, distinctive vistas, and captivating narratives. The novel viewpoint of healthcare in tourism will foster new business opportunities and advance tourism innovation. Additionally, it is important to well understanding the relationship between tourism sites, travel trends and individuals’ self-identification, which would provide more detail leisure and tourism market the strengthen the linkage between destinations and visitors.

For politicians, the City Walk presents a chance to enhance city tourism and create a new travel trend that appeals to youthful people with greater market potential. The policymaker’s comprehension of the motive behind the emergence of new travel trends compels them to implement measures that involve guiding, facilitating, and establishing diverse sites of interest for city tours, with the aim of enhancing the long-term viability of city tourism. In addition, the expansion of City Walk would compel the local authorities to enhance the accessibility of the city, rendering it more easily reachable and welcoming to visitors. This would be advantageous for both the local inhabitants and tourists. Furthermore, advocating for the City Walk tour inside the city would be very pertinent to the advancement of both individual and municipal health. Developing pedestrian-friendly leisure and tourism destinations, generating more employment opportunities, and fostering a harmonious connection between the tourism industry and the local community. Thirdly, this study would prompt policymakers to enhance their understanding of emerging travel trends and reassess the scope of city tourism strategies by promoting the integration of leisure and tourism, dissolving the boundaries of city tourism sites, and categorizing more specific markets that cater to the diverse needs of visitors.

#### Limitations and future research.

While this study offers valuable insights, it is crucial to acknowledge its distinct limitations that might guide future research directions. The primary methodology utilized for data collection was a quantitative approach, via self-administered questionnaires. To enhance understanding, future research should explore qualitative or mixed-method approaches, employing a range of tools for data collection. By implementing this broad technique, there is a possibility of gaining a more nuanced understanding of the elements that influence City Walk.

Future study should also explore many areas to examine the specific types of social influence that have either positive or negative impacts on self-identification. The absence of any impact on self-identification is due to the lack of variety seeking in City Walk. If there are any more elements that might contribute to the purpose behind tracking travel trends. Moreover, it is imperative for future research to ascertain efficacious strategies for elucidating the diverse array of emerging travel trends in the academic realm. This will facilitate a comprehensive comprehension and accurate interpretation of these novel occurrences, ultimately fostering robust methodologies to bolster urban tourism growth. Furthermore, it is crucial to prioritize acquiring a deeper understanding of every subject through additional research. By addressing these subjects, we can enhance our comprehension of City walk in the context of City tourism and develop a more comprehensive and comparative understanding. This will enable us to construct more effective plans for the expansion of city tourist.

## Supporting information

S1 File Questionnaire. (DOC)

S2 File Data. (XLSX)
