# Supplementary material for: Why are individuals tracing travel trends? A case study of City Walk in Malaysia
Source: PLoS One. 2025 Feb 10;20(2):e0309493. doi: 10.1371/journal.pone.0309493 (PMC11809927; doi:10.1371/journal.pone.0309493)
Supplement: S1 File — (DOC) [file pone.0309493.s001.doc]

Questionnaire

Respondent No:

Why are individuals tracing travel trends? A case study of City Walk in Malaysia

In this questionnaire, you will be asked opinions related to explore the motivation of tourism to follow travel city walk in Malaysia. This research aims to provide insight to better understand the motivation of tourists’ behavioral intention in City walk in Malaysia. Your response will be greatly appreciated.

The results of this survey will only be published in aggregate form. The information provide will be treated as strictly confidential and no data about you will be released. Your completion of this questionnaire will be taken as consent to participate in this study. This questionnaire will take you approximately 3 to 5 minutes to complete.

Your cooperation is highly appreciated.

**Section A**

Demographic Profile：

Please indicate the extent to which you agree with the following statements by ticking (√) the appropriate response.

D1. To which city you have been for city walk in Malaysia in recent one year

□Kuala Lumpur □TMelaka □Pulau Penang □Kuching □Ipoh

□Others (Please specify)_______

D2. Your nationalisty:_______

D3. Gender: □Male □Female

D4. Age：（Please specify）:_________

D5. Level of Education:

□Some high school □High school graduate □Some college

□College graduate □Some graduate school □Completed graduated school □Others（please specify）：————

D6. Occupation:

□Management □Technical worker □Government □Professional

□Agricultural worker □Self- employed □Service worker □Housewife

□Student □Retired/Not in workforce □Others, (please specify）_______

D7. How often do you participate in city walk:

□Daily □Weekly □Monthly □Rarely □Never

D8. On average, how long is your typical city walk?

□Less than 1 hour □1-3 hours □4-6 hours □7-9 hours □More than 9 hours

D9. How do you ususally travel to the starting point of your city walks?

□Walk □Bicycle □Public Transport □Car □Other (please specify):____

D10. WIth whom do you usually participate in city walk?

□Alone □With family □With Friends □WIth a tour group □Other (please specify):______

**Section B:** On the scale of 1-5. Please indicate the extent to which you agree with the following statements by ticking (√) the appropriate response. 1 is strongly disagree, 5 is strongly agree.

Over the past month, how often have you experiened the following feelings while participating in city walks? (use the scale where 1= never, 2= rarely; 3= sometimes, 4= often, 5= always)

| **Self identification** | 1 | 2 | 3 | 4 | 5 |
| --- | --- | --- | --- | --- | --- |
| The city walk reflects who I am |  |  |  |  |  |
| I can identify with city walk. |  |  |  |  |  |
| I feel a personal connection to city walk |  |  |  |  |  |
| I think city walk to be “me ”(It reflects who I consider myself to be or the way that I want to present myself to others) |  |  |  |  |  |
| City walk suit me well |  |  |  |  |  |
| I use city walk communicate who I am to other people. |  |  |  |  |  |
| Variety seeking |  |  |  |  |  |
| When I travel out, I like to try the most unusual items, even if I am not sure I would like them. |  |  |  |  |  |
| While preparing traveling, I like to try out new routines. |  |  |  |  |  |
| I think it is fun to try try out new items one is not familiar with. |  |  |  |  |  |
| I am eager to know what kind of charm from other areas. |  |  |  |  |  |
| I like to experience exotic charm. |  |  |  |  |  |
| Items in different areas that I am unfamiliar with make me curious. |  |  |  |  |  |
| I prefer to experience tourism products or activities I am used to. |  |  |  |  |  |
| I am curious about tourism products I am not familiar with. |  |  |  |  |  |

**Health Care**

| Mental heathy | 1 | 2 | 3 | 4 | 5 |
| --- | --- | --- | --- | --- | --- |
| Feeling happy and content. |  |  |  |  |  |
| Feeling relaxed and stress-free |  |  |  |  |  |
| Feeling more energetic |  |  |  |  |  |
| Feeling a sense of adventure and excitement. |  |  |  |  |  |
| Feeling more sociable and less lonely. |  |  |  |  |  |
| Feeling a sense of personal accomplishment |  |  |  |  |  |
| Physical health assessment | 1 | 2 | 3 | 4 | 5 |
| Feeling ohysically active and fit. |  |  |  |  |  |
| Experiencing less physical pain or discomfort. |  |  |  |  |  |
| Having improved sleep quality. |  |  |  |  |  |
| Feeling physically refreshed and rejvvenated. |  |  |  |  |  |
| Experiencing improved appetite. |  |  |  |  |  |
| Having fewer instances of illness or phycial ailments. |  |  |  |  |  |
| Feeling stronger and more physically capable. |  |  |  |  |  |

**Social influence**

| Normative social influence | 1 | 2 | 3 | 4 | 5 |
| --- | --- | --- | --- | --- | --- |
| I often join city walks recommended by my friends or family |  |  |  |  |  |
| I choose city walk routes based on where my peers have gone |  |  |  |  |  |
| I feel encouraged to join city walks that are popular among people I know. |  |  |  |  |  |
| I prefer to join city walks that are trending on social media. |  |  |  |  |  |
| Informational social influence | 1 | 2 | 3 | 4 | 5 |
| I rely on reviews and ratings when choosing city walks. |  |  |  |  |  |
| I often ask for advice from friends or family before describing on a city walk route. |  |  |  |  |  |
| I trust the opinions of city walk bloggers and influencers when planning my walks. |  |  |  |  |  |
| I use social platforms to get ideas for my city walk itineraties |  |  |  |  |  |
| Identification with social group | 1 | 2 | 3 | 4 | 5 |
| I like to participate in city walks that reflect my social status. |  |  |  |  |  |
| I choose city walk routes that I belives will impress others |  |  |  |  |  |
| My participation in city walks is influenced by the places visited by people I admire. |  |  |  |  |  |
| I like to share my city walk experiecnes on social media to gain social approavel. |  |  |  |  |  |

**Behavioral Intention**

| For the city walk | 1 | 2 | 3 | 4 | 5 |
| --- | --- | --- | --- | --- | --- |
| I will speak well about city walk. |  |  |  |  |  |
| I will recommend city walk if someone asks for my advice |  |  |  |  |  |
| I will encourage my friends, relatives and colleages to participate city walk |  |  |  |  |  |
| In future I intent to participate in city walk. |  |  |  |  |  |

--- The End ----
